# Navigating social and ethical challenges of biobanking for human microbiome research

**DOI:** 10.1186/s12910-016-0160-y

**Published:** 2017-01-11

**Authors:** Kim H. Chuong, David M. Hwang, D. Elizabeth Tullis, Valerie J. Waters, Yvonne C. W. Yau, David S. Guttman, Kieran C. O’Doherty

**Affiliations:** 1 Department of Psychology, University of Guelph, Guelph, ON N1G 2W1, Canada; 2 Department of Laboratory Medicine & Pathobiology, University of Toronto, Toronto, Canada; 3 University Health Network, Toronto, Canada; 4 Adult Cystic Fibrosis, University of Toronto, Toronto, Canada; 5 Toronto Adult Cystic Fibrosis Centre, St Michael’s Hospital, Toronto, Canada; 6 Department of Paediatrics, University of Toronto, Toronto, Canada; 7 Division of Infectious Diseases, Hospital for Sick Children, Toronto, Canada; 8 Department of Paediatric Laboratory Medicine, Hospital for Sick Children, Toronto, Canada; 9 Department of Cell & Systems Biology, Centre for the Analysis of Genome Evolution & Function, University of Toronto, Toronto, Canada

**Keywords:** Return of results, Incidental findings, Identifiability, Biobank, Microbiome, Cystic fibrosis, Research ethics

## Abstract

**Background:**

Biobanks are considered to be key infrastructures for research development and have generated a lot of debate about their ethical, legal and social implications (ELSI). While the focus has been on human genomic research, rapid advances in human microbiome research further complicate the debate.

**Discussion:**

We draw on two cystic fibrosis biobanks in Toronto, Canada, to illustrate our points. The biobanks have been established to facilitate sample and data sharing for research into the link between disease progression and microbial dynamics in the lungs of pediatric and adult patients. We begin by providing an overview of some of the ELSI associated with human microbiome research, particularly on the implications for the broader society. We then discuss ethical considerations regarding the identifiability of samples biobanked for human microbiome research, and examine the issue of return of results and incidental findings. We argue that, for the purposes of research ethics oversight, human microbiome research samples should be treated with the same privacy considerations as human tissues samples. We also suggest that returning individual microbiome-related findings could provide a powerful clinical tool for care management, but highlight the need for a more grounded understanding of contextual factors that may be unique to human microbiome research.

**Conclusions:**

We revisit the ELSI of biobanking and consider the impact that human microbiome research might have. Our discussion focuses on identifiability of human microbiome research samples, and return of research results and incidental findings for clinical management.

## Background

Literature on biobanks has mainly focused on human genetics and genomics, with many ethical challenges remaining unresolved [1, 2]. Rapid advances in human microbiome research can further complicate matters. This field of research aims to elucidate the role of bacteria and other microbes in different areas of our body in health maintenance and disease development. In this paper, we discuss the ethical, legal and social implications (ELSI) related to biobanking for human microbiome research. We draw on two cystic fibrosis (CF) biobanks located in Toronto, Canada, to illustrate our points where applicable: the Toronto CF lung microbiome biobank housed at the Toronto General Hospital and the CF sputum biobank at the Hospital for Sick Children. We focus specifically on: 1) ethical considerations of categorization and identifiability of samples biobanked for human microbiome research; and 2) clinical actionability of human microbiome research and ethical challenges associated with returning individual research results and incidental findings.

CF is a common fatal genetic disease among individuals of European backgrounds, with an estimated frequency of about 1 in 2500 live births [3]. Patients experience progressive deterioration of pulmonary function and eventually death due to recurrent microbial infections of the respiratory tracts. Although substantial advances in clinical care and antibiotic treatments have increased life expectancy over the past few decades, with the median survival age in Canada currently at 49 years [4], patient health deteriorates in spite of seemingly appropriate treatments. The two CF biobanks have been established as publicly available repositories to facilitate sample and data sharing for research into CF progression, including characterizing the diversity and dynamics of the microbial communities in the lungs of pediatric and adult patients. The long-term goal is to facilitate the development of effective and tailored treatments that can control lung infections.

We begin by providing an overview regarding some of the ELSI of human microbiome research. Given that the human microbiome can be changed by personal lifestyle and environmental conditions, there has been considerable scientific and public interest in the modification of the human microbiome for health and disease management. However, modifying the human microbiome for health-related reasons may have social and ethical implications for the individuals, their family members and even broader communities. We then explore the implications of human microbiome research for the conceptualization of the human being and human identity, and outline ethical considerations regarding the identifiability of samples biobanked for human microbiome research (e.g., sputum). We propose that samples collected from microbiome research participants should be treated with the same privacy and confidentiality safeguards as human tissue samples and other identifying sources of information. We refer to such samples as human microbiome research samples, which we differentiate from bacterial cultures that consist of bacterial strains isolated from these samples, grown in the laboratory, and cannot be linked back to the donors. Finally, we examine the issue of return of research results and incidental findings for human microbiome research in general, and for disease-specific biobanks, such as the CF biobanks, in particular. We suggest that the return of individual findings could provide a powerful clinical tool for care management as the dynamic nature of the human microbiome may offer substantial opportunity for intervention and health modulation. We outline the ethical challenges associated with returning individual findings and highlight the need for a more grounded understanding of contextual factors that may be unique to human microbiome research.

### Overview of human microbiome research

The Human Microbiome Project (HMP) was launched by the U.S. National Institutes of Health in 2007, and included a number of member organizations and repositories connecting to a central data repository known as the Data Analysis and Coordination Center (DACC). A central aim has been to characterize a “core” human microbiome, using healthy adult cohorts, that is common to all or a vast majority of humans across several body regions, including the nasal passages, oral cavity, skin, gastrointestinal tract and urogenital tract [5, 6]. Findings about the high variability of microbial composition across individuals and across body sites within an individual have led to further research into the stability of the human microbiome over time and the effects of environment on its composition. While the initiative has produced valuable scientific knowledge, some HMP researchers have questioned the representativeness of the sample populations and what it means to be “normal” and “healthy” [7]. The extensive sampling with Euro-Americans of middle to upper socioeconomic status has raised social justice concerns that science may be more prepared to develop interventions with this group than other social groups [8]. It also raises the question of how the HMP may be ethically promoted to underserved populations, who may have high hopes for therapeutic benefits or are vulnerable to commercial exploitation.

There has been a surge of basic and translational research to investigate the mechanisms of the human microbiome and how it may be modulated to improve health and avoid disease. Most research efforts have focused on the vital functions of the gut microbiota in the maintenance of homeostasis in the body, with promising insights into the etiology, diagnosis and treatment of various health problems, including gastrointestinal diseases, colon cancer, types 2 diabetes and obesity [9–11]. In the human gut, the resident microbes carry out an array of metabolic activities that are distinct from those encoded by the human genome but are essential for bodily functioning. These metabolic activities have major consequences that can be beneficial or harmful. Gut microbes help break down nondigestible dietary products and contribute nutrients and energy to the human body [9]. A balance is thus maintained with the human host’s metabolism and immune system. A disruption in the gut microbiota can lead to inflammation and infection, and contribute to the development of gastrointestinal diseases and possibly diabetes and obesity. Translational research efforts have begun to explore alternative interventions for treating gastrointestinal and other types of disease, which emphasize the maintenance of a healthy microbiome rather than the eradication of bacteria by antibiotics. These include the use of diets [12, 13], probiotics and prebiotics [14–16], and microbial ecosystem therapeutics [17].

Advances in human microbiome research have raised high hopes about potential medical applications along with cautions about scientific validity, clinical utility, and social and ethical challenges. Many of the prevailing ethical issues in human microbiome research overlap with those in human genetic research. The ELSI literature on human microbiome research has largely focused on informed consent mechanisms, return of research results and incidental findings to participants, data sharing, privacy and confidentiality, ownership of samples and benefit sharing which have implications for social justice issues [7, 18–21]. There has been limited study into broader ELSI with regard to the public impact of human microbiome research. For example, Slashinski et al. examined investigator perspectives on the commercialization of human microbiome research [22]. Their study raised concerns over how the scientific value of research into the human microbiome and health is mobilized by the marketplace to promote the consumption of probiotics and dietary supplements, despite a lack of regulation and knowledge about the safety and effectiveness of these products. The authors suggested “the need to find a balance between the marketplace, scientific research, and the public’s health” [p.1].

Efforts to translate human microbiome research into new medical applications and technologies create important opportunities and challenges scientifically and socially. The knowledge that an individual’s microbiome can be modified by personal lifestyle and environmental conditions is leading to promising health interventions, as well as concerns about the social and ethical implications of such purposeful modification. Sonnenburg and Fischbach discussed therapeutic opportunities created by manipulating the human microbiome, such as through probiotics, prebiotics, and small-molecule and biological drugs [23]. The application of diets and probiotics to improve health and manage disease has captured considerable attention from the public, especially for gastrointestinal diseases. However, a review of online dietary recommendations and popular dietary regimes for inflammatory bowel disease found conflicting information and a lack of evidence-based guidelines [24]. Furthermore, probiotics are classified as food or dietary supplements and do not undergo a vigorous regulatory evaluation, which can result in variable product quality and unsubstantiated claims about their therapeutic benefits [23]. Petrof et al. examined the application of microbial ecosystem therapeutics, including fecal transplants and synthetic stool, to restore a patient’s disrupted gut microbiota [17]. Fecal transplants involve introducing the stool from a healthy donor into the gastrointestinal tract or colon of the patient, and have been reported to be a successful treatment for *Clostridium difficile* infection (CDI) and gastrointestinal diseases [17, 25–27]. However, the treatment has not gained traction in medical institutions, which could be due to a lack of knowledge about safety and long-term outcomes. There have also been suggestions about the treatment’s unappealing nature as a barrier to its uptake. In Canadian media, fecal transplants have often been portrayed as being inherently disgusting [28]. Termed the “ick factor,” the allegedly disgusting nature of the treatment is used to construct different messages about its social acceptability and status as a legitimate treatment. CDI and gastrointestinal diseases pose significant economic and social burdens to affected patients, their families, and health care systems in many developed countries. In Canada, regulatory clarity surrounding fecal transplants had been lacking for many years. It was not until March of 2015 that Health Canada drafted a guidance document to classify fecal transplants as a biological drug and approve its use for the treatment of CDI not responsive to conventional therapies [29]. Use for other medical conditions is still under the regulations of an investigational clinical trial. The case examples of probiotics and fecal transplants illustrate the need to examine public understandings of and commercial interests in human microbiome research and related medical applications.

O’Doherty, Virani, and Wilcox have argued that social and ethical implications may arise from technologies to modify the human microbiome, which can go beyond the individuals [30]. The composition and diversity of the human microbiome can change as a result of lifestyles and environmental conditions that are associated with broader sociocultural practices. Societal shifts to a more sedentary lifestyle and an industrialized diet rich in animal proteins, fats and simple carbohydrates in Western countries have a negative impact on the gut microbiota diversity [12, 13, 31]. A Westernized diet has been associated with increasing risks of developing obesity, gastrointestinal diseases, cardiovascular disease and colon cancer. Moreover, there is evidence that the microbiota compositions at different body sites are similar between family members and people in regular close contact [32, 33], suggesting that microbiomes can be transmitted and shared between individuals. Interestingly, a recent experimental study found that a reduction in gut microbiota diversity was also observed for the housemates of participants who took amoxicillin or azithromycin, two commonly prescribed antibiotics [34].

The notion that changes in the human microbiome can be affected by other individuals raise important social and ethical considerations. At an individual level, questions arise about the impact that an individual’s decision to modify his or her own microbiome may have on other individuals with important implications for autonomy [30]. Potentially, an individual’s microbiome may be affected by the health decisions of others without his or her awareness. At a societal level, there are public health implications if a large number of individuals adopt a health practice to change their microbiomes. With rapid advances in human microbiome research, it is also conceivable to design public health initiatives that involve the purposeful modification of the human microbiome to yield health benefits to the broader society. Such large-scale initiatives might bypass individual health decision-making and bring about unanticipated and long-term consequences of changing the microbiomes of many individuals in a community (See O’Doherty et al. for more details on public health implications of technologies developed on the basis of human microbiome research) [30].

In the sections below, we focus our discussion on the ethical implications of biobanking for human microbiome research that can have profound impact on the individuals. In particular, we provide a detailed discussion with regard to whether microbiome-related data can provide additional information that is personal and sensitive beyond genetic data and the potential of returning research findings for clinical care.

### Categorization and identifiability of the human microbiome

In this section, we focus on ethical issues associated with categorizing human microbiome research samples and the debate over whether the human microbiome can provide uniquely identifying information. Advances in human microbiome research have been argued from an ecological and evolutionary perspective to have dramatic implications for how we should conceptualize the human being. Dethlefsen, McFall-Ngai, and Relman explored the human-microbe symbiotic relationship and how microbes and their human hosts have co-evolved and selected for mutualistic interactions that are essential for human health [35]. It has been argued that humans are complex “superorganisms” who possess an “extended genome” of millions of microbial genes [10]. Rhodes asserted that the concept of humans as “superorganisms” recasts the view of humans from “atomistic individual organisms” to “an amalgam of us and them,” and “our coexistence with the microbiome tells us that human evolution is not just human history” ([36], p.2). O’Hara and Shanahan described the gut microbiota as a “forgotten organ” given that it has “a metabolic activity equal to a virtual organ within an organ” ([37], p.688). Sonnenburg and Fischbach also stated that “our microbiota is more like an organ than an accessory: These microbes are not just key contributors to human health but a fundamental component of human physiology” ([23], p.1).

Despite scientific conceptualizations of human beings as superorganisms, it is difficult to determine the uptake of this perspective in our sense of self in everyday life. Gligorov and colleagues argued that, to the extent that it permeates everyday life, scientific knowledge of the human microbiome may have an impact on how we think of ourselves and how we perceive ourselves in relation to the environment and other people, which can influence our sense of moral responsibilities and behaviors [38]. They pointed to the shifts in the conceptualization of the human self that have stemmed from past scientific discoveries. These include the discoveries that mental activities are located in the brain and the existence of neurotransmitters, and the mapping of the human genome and association of DNA with human traits and mental illnesses. Messages about the human body/microbe ecosystem have become more accessible to the public in recent years through the media. Nerlich and Hellsten suggested that metaphorical framings in the media since 2003 onwards have personified microbes as having a quasi-human agency and emphasized the interactions between microbes and between microbes and humans [39]. In these media framings, microbes are no longer conceptualized solely as enemies and a danger to health but also friends and healers. Humans are also framed as hybrids that blur the boundaries between the human self and microbes.

From a bioethical perspective in the context of research and biobanking, it is currently not clear whether the human microbiome should be categorized as part of or separate from the human body [18]. Similar to some samples collected and biobanked for human genetic and other research purposes, some types of samples biobanked for human microbiome research might otherwise be considered waste (e.g., dead skin, feces, saliva). In the case of the Toronto CF lung microbiome biobank, sputum samples from the St. Michael’s Hospital Adult CF Clinic are collected from adult patients who have consented to participating in the Toronto CF Lung Microbiome Team’s study and to providing an extra sputum sample at each clinic visit. This sample is collected in addition to those provided as part of routine clinical care. In contrast, pediatric CF sputum samples collected at the Hospital for Sick Children represent excess material from specimens collected for routine microbiologic testing, which would otherwise have been discarded following completion of testing. Assent from pediatric patients and consent from parents are obtained for biobanking the excess material for research purposes.

The fact that there are opportunistic bacteria and other microbes which are harmful, and potentially fatal, forms the ontological basis for the traditional view of microbes as pathogens and inimical intruders separated from the human body. On the other hand, it could also be argued from an ecological and evolutionary perspective that the human microbiome is *part of* the human being due to the co-evolution of humans and microbes and the vital bodily functions performed by microbes. The description of the gut microbiota as an “organ” situates the gut microbial communities collectively as part of the human body. It could, thus, be argued that samples collected for human microbiome research should be treated as human tissue samples (e.g., tumours and blood).

In addition to microbes, human microbiome research samples generally contain human cells and/or DNA. The HMP has stipulated that metagenomic data are deposited into a public open-access database while potentially identifying data (e.g., clinical data, human DNA data) are placed in a controlled-access database [40]. An interview study with HMP investigators reported that, although some investigators felt that participants’ privacy and confidentiality were protected, others expressed concerns over the inadvertent release of human DNA to the public database as a result of inadequate filtering methods, computational limitations, or unmapped portions of the human genome [20]. Improvements in filtering methods and new understandings of the human genome can mitigate the concern about human DNA data contamination in metagenomic data. However, the issue that human microbiome research samples contain human DNA still raises concerns about privacy and confidentiality since these samples can be analyzed in ways that are identifiable. In this respect, we suggest that human microbiome research samples should be treated by biobanks with the same safeguards in terms of privacy and confidentiality as any other human tissue samples or identifying sources of information. However, we draw attention to circumstances where researchers obtain samples from a biobank to culture bacteria for research purposes without having any contact with the donors. If the bacterial cultures exist separately from donor samples and cannot be linked back to the donors, then a strong argument can be made for not considering bacterial cultures that consist of specific bacterial strains isolated from donor samples and grown in the laboratory to be “human” samples.

Wolf et al. used the term, “biobank research system,” to illustrate the relationship between three entities: 1) primary researchers or collection sites feeding data and samples into the biobank; 2) the biobank, with some biobanks collecting and analyzing data/samples themselves; and 3) secondary researchers accessing data/samples from the biobank for further research [41]. Arguably, a biobank for human microbiome research has the management responsibility to ensure that secondary researchers who receive the coded data and/or samples cannot readily re-identify the subjects, if the design of the biobank has been established to maintain the possibility of re-identification. Samples and data may have already been de-identified by the primary researchers or collection sites prior to being sent to the biobank. The two Toronto CF biobanks and accompanying database of clinical metadata are subject to management regulations that are common practices across many biobanks. According to Petersen and Van Ness, some centralized biobanks do not retain identifiers or contact information of donors to both protect privacy and avoid the consideration of returning research results [42]. Since the long-term goal of the CF biobanks is to facilitate the development of tailored treatments that can control lung infections, they have been set up to retain the possibility of re-identification of patients and linkage to clinical data. All specimens are identified only by arbitrarily assigned patient study ID and specimen ID numbers. Patient identity (including identifiers such as medical record numbers) for each study ID number is known only to the clinic coordinators who interact with patients during clinic visits, and to the principal investigators who oversee specimen collection at each site. Any other investigators are able to access only the study and specimen ID numbers. Occasional situations have arisen necessitating re-identification of specific patients, usually relating to the need to link study ID numbers for patients whose clinical care has been transferred from the pediatric CF clinic (Hospital for Sick Children) to the adult CF clinic (St. Michael’s Hospital), or to link sputum samples to de-identified lung tissue obtained from the same patient following lung transplantation (Toronto General Hospital). In such situations, the initial study ID is submitted to the principal investigator overseeing specimen collection at the originating clinic, who then communicates patient identity directly (and only) with the principal investigator for the receiving clinic or lung transplant program. The receiving principal investigator links the patient identity to the appropriate study ID number and communicates only the study ID number to secondary investigator(s) requesting the linkage, who are then able to use the additional study ID numbers to access additional specimens and/or clinical data.

At present, there is a lack of scientific consensus over whether microbiome-related data is uniquely identifying like human genetic data. There have been some research findings that suggest the potential of these data to identify group affiliation and more personal information. Arumugam et al. identified three enterotypes or clusters of human gut microbiome based on the clustering of key bacterial genera from 39 samples involving six nationalities and including 22 sequenced fecal metagenomes of European individuals [43]. Sequencing of dental plaque and saliva samples from 192 participants of four ethnicities in the United States (African, Caucasian, Chinese, and Latino) found ethnic-specific clustering of microbial communities [44]. However, Jeffery, Claesson, O’Toole and Shanahan commented that there is considerable debate about, and blurring of, the notion of distinct enterotypes of the gut microbiota, and such categorization may have oversimplified the complexity [45]. At an individual level, it has been reported that skin-associated bacteria recovered from the surfaces of computer keyboards and mice could be used as microbial “fingerprints” to identify the individuals, and microbial fingerprint analysis could present a valuable resource for forensic identification [46]. However, it is difficult to evaluate the practical utility of information from microbial fingerprints giving that the human microbiome is subject to modification by lifestyles and environmental factors. The scientific uncertainty over the stability of the microbiome raises questions about whether microbial fingerprints can still be linked to an individual after a certain amount of time [18].

It is possible that the information gleaned from an individual’s microbial profile at a specific point is not identifying at a later point in time. However, this does not preclude the possibility of microbial profiles being combined with genetic and other types of information in ways that are more personally revealing, such as providing additional information about past exposures, visits to other countries, predisposition to certain conditions, sexual practices, diet, and consumption of tobacco, alcohol and other drugs. The information could potentially be used in ways that are detrimental to the persons involved. Hoffmann et al. have raised the issue that both human genetic and human microbiome research may bring into existence new group stigmas that are unknown to researchers or individuals prior to participation in the research [7]. Of concern is whether existing laws and regulations, such as the Personal Information Protection and Electronic Documents Act (PIPEDA) in Canada or the Genetic Information Nondiscrimination Act (GINA) in the U.S.A., are adequate and sufficient to protect individuals or groups from stigmatization and discrimination based on information from their microbiome.

### Return of individual research results and incidental findings

Despite significant advances in human microbiome research, there is limited understanding of the practical and clinical relevance of most research findings to date. Knowledge of an individual’s microbial profile could potentially open up more decision points for diagnosis, treatment, and risk management [18]. Unlike the human genome which remains relatively static throughout an individual’s life, the dynamic nature of the human microbiome could provide substantial opportunity for intervention and microbial data could be more clinically actionable than human genetic data. The translation of human microbiome research into practical approaches for diagnosis and treatment has generated extensive interest in the scientific community. Zakutar and colleagues demonstrated that analysis of the gut microbiome could potentially be used with other known clinical risk factors as a screening tool to improve early detection of colorectal cancer [47]. Dominguez-Bello et al. conducted a pilot study in which four newborns delivered by caesarean section were exposed to maternal vaginal fluids at birth [48]. They found that the gut, oral, and skin microbial communities of these newborns were enriched and comparable to vaginally delivered babies. Although the long-term health effects are unknown, the study demonstrates the potential to restore vaginal microbes in C-section delivered newborns, who are at a higher risk of developing obesity, asthma, allergies and other immune deficiencies. In CF patients, *Pseudomonas aeruginosa* and species of the *Burkholderia cepacia complex* (BCC) are among the most common bacterial pathogens isolated from lung infections. They are often implicated in acute episodes of respiratory decompensation and in acceleration of pulmonary deterioration. Coburn et al. found that microbial diversity and lung function are greater in pediatric patients and lower in older age groups, with lower diversity correlates with worse lung function [49]. Hampton et al. also reported that microbial diversity is greater in pediatric patients and microbial communities in pediatric patients living together are more alike than those living apart [50]. They proposed that, since the environment appears to be an important determinant of the microbiota in pediatric patients, early intervention to maintain or enhance its diversity may hold promise for slowing disease progression. Effective intervention and disease management require a clearer understanding of how the airway microbial communities in CF patients evolve and how they are influenced by repeated antibiotic treatments and other environmental factors.

For CF patients with end-stage lung disease, lung transplantation offers the only viable option. But a relatively scarce supply of donor organs limits its availability. Many transplant centers exclude patients with BCC infection from the waiting list since they are at a greater risk for post-transplant complications and have a higher mortality rate than patients without the infection. More recent studies have indicated that the impact of BCC infection varies by different species and strains. Patients with BCC infection were regularly transplanted at the Duke University Medical Center in Durham, US, until 2002 [51]. A retrospective review of 75 patients undergoing lung transplantation from 1992 to 2002 at the center indicated that the 1-year and 5-year survival rates of patients infected with the *B. cenocepacia* species were significantly lower compared to patients infected with other BCC species and non-infected patients. Likewise, a retrospective review of the lung transplantation database at the Freeman Hospital, UK, from 1989 to 2010 found that patients with *B. cenocepacia* infection had significantly higher post-transplant mortality rate, resulting in the center no longer accepting patients with this condition on the waiting list [52]. The Toronto Lung Transplant Program is a notable exception for not excluding patients with BCC infection. At present, microbial sequencing poses no risk to patients enrolled in the program in terms of being disqualified for lung transplantation as a result of bacterial detection in their samples. However, questions regarding ethical obligations in similar situations could arise if individual research results or unexpected incidental findings could potentially lead to significant alterations in disease management.

The bioethics literature is rich with debate about the ethical obligations of researchers, if any, to disclose research results or incidental findings and how disclosure should be managed. Generally speaking, offering to return aggregated research results to participants is considered to be an ethical research practice. More controversial is the issue of whether participants should receive feedback on their individual data. The debate over returning individual findings has mainly focused on human genetic and human genomic research. Bredenoord et al. asserted that the majority of perspectives in the literature on returning individual genetic results advocate for either a very restrictive disclosure, wherein individual results should not be returned except for life-saving data, or an intermediate position of qualified disclosure, which holds that results should be disclosed if they meet particular conditions [53]. The authors suggested that, rather than focusing on whether research results should be returned, the debate should address how to strike a balance between the benefits and harms of disclosure and the appropriate procedures for deciding.

Defining the criteria for returning research results remains a matter of debate and may vary with research contexts. Burke, Evans, and Jarvik indicated that efforts should be made to clarify the criteria for returning results, appropriate caveats, and appropriate referrals if the information is clinically relevant [54]. Likewise, Rahimzadeh and colleagues argued for the need to recognize contextual factors in determining the return of incidental findings in pediatric oncology genetic research [55]. Their interview study with 16 investigators identified four key considerations for determining when and under what circumstances to return. These include 1) clinical significance of results with clinical actionability being one of the foremost reasons for disclosure; 2) respect for individuals; 3) scope of professional responsibilities and the need to take into account researcher/clinician expertise and potential for liability; and 4) implications for the healthcare/research system including funding duration, resource constraints, and availability of professional expertise such as genetic counselling. According to Rahimzadeh et al., a one-size-fits-all approach may risk overlooking relevant contextual factors.

The role that biobanks may play in human microbiome research is still unclear due to the relative infancy of the field and the lack of regulations regarding biobank management and governance [56]. In the context of human genomic research, Wolf et al. recommended that, rather than placing all the responsibilities on primary researchers or collection sites, biobanks have significant responsibilities for the planning and management of return of research results and incidental findings [41]. The authors suggested that findings that are analytically valid, reveal an established and substantial risk of a serious health condition, and are clinically actionable, should be returned to consenting participants. As such, the coordination and allocation of responsibilities between the biobanks, primary researchers/collection sites, and secondary researchers should be made explicit. Managing return of results would require resources and appropriate processes to realize the benefits of disclosure without impairing the primary scientific purpose of biobanks [41, 42]. These include dedicated personnel, clinical expertise and clinical referral information, and resources to securely maintain identifying and follow-up contact information. Many questions remain about the practicality and cost constraints of returning individual results.

### Clinical significance and actionability of human microbiome research

In human microbiome research, concerns have been raised that data may be interpreted prematurely or incorrectly [7, 18–20]. Typically, before research findings can be used in clinical care, further establishment of scientific validity, reliability, and clinical significance is needed. Presently, sequencing of sputum samples at the two CF biobanks often occurs long after sample collection and the impact of microbiome-related data on clinical outcomes is almost completely unknown. This does not, however, preclude the possibility that research or incidental findings could be clinically actionable in the future. It has been suggested that effective early intervention to maintain or enhance the microbial diversity of CF pediatric patients may improve health and slow disease progression [50]. Therefore, the return of individual microbiome findings could provide a powerful clinical tool for CF patient care management. However, a necessary condition for the return of individual results is that data can be linked back to a specific participant. Effective translation of research into clinical care also requires the flow of information between the biobank, primary and secondary researchers, and the clinical team, which may potentially increase the risks to privacy and confidentiality. For disease-specific biobanks like the CF biobanks, a communication plan should be established with secondary researchers in the event that research or unexpected incidental findings are significant for clinical care, or the plan can be included in the Material and Data Access Agreement. The biobanks will need appropriate procedures in place to deal with re-identification, if they engage in the processes of disclosure, and have the appropriate consultation and policy-making capacity to determine under what circumstances to return and what evaluation criteria should be used to make the decision [41].

The questions of whether, how, and under what contexts to return research or incidental findings have led to much discussion in both human genetic and human microbiome research [7, 18–20]. The lack of knowledge about the clinical significance of most research findings proves to be challenging for disclosure, which can cause psychological or social harm if findings are misinterpreted or inadequately interpreted. The possibility of returning individual results also raises questions of whether and how this should be addressed in the consent processes in a way that participants can understand without overstating the benefits of participation or raising false expectations of therapeutic benefits. Moreover, in human microbiome research, incidental findings of asymptomatic or subclinical infectious disease raise not only the need for disclosure to participants, but potentially to public health authorities and other people who may be affected [7, 19]. This is complicated by the issue that clinical manifestation may depend on a number of factors, including interactions between the infecting species with other microbes and the immune system, genetics, age, and lifestyles. Hoffman and colleagues asked the following questions: “Should the disclosure determination be based on clinically significant findings even if the individual is asymptomatic of the condition found? If so, do we know what constitutes clinically significant pathology in terms of our microbiome?” ([7], p. 466). Knowledge of being at risk of developing a serious health condition could cause anxiety and distress, especially if there is no known cure or treatment. It could also lead to adverse social consequences. In bioethics, the principle of non-maleficence requires researchers to not create unnecessary harm or injury, either through acts of commission or omission. The unclear delineation between bacterial colonization and infection poses the ethical dilemma of assessing under what circumstances and to whom to disclose, which requires a careful balance of the potential benefits and harms of disclosing compared to not disclosing.

In human genetics and human genomics, it has been recommended that research results and incidental findings that are analytically valid, clinically significant and actionable should be returned to participants who have consented to receiving research findings [41, 55]. While these recommendations are relevant for human microbiome research, there is a need for more grounded understanding of contextual factors that may be unique to human microbiome research in addressing what criteria to determine returnable findings and developing ethical practices for managing disclosure. Two survey studies with American institutional review board (IRB) and Canadian research ethics board (REB) chairs and vice-chairs, respectively, found that most respondents saw a prominent role for IRBs/REBs in defining the criteria, circumstances, and processes by which individual genetic research results should be returned to participants and their families [57, 58]. Likewise, the roles of IRBs/REBs, researchers, biobank personnel, and research participants should be further deliberated in human microbiome research to develop ethical guidelines and practices with respect to return of results.

## Conclusions

We have discussed the social and ethical challenges associated with biobanking for human microbiome research, focusing on the debate over identifiability and ethical challenges of returning individual results and incidental findings for clinical management. We suggest that human microbiome research samples should be treated with the same privacy and confidentiality safeguards as human tissue samples or other identifying sources of information, not only because of the possibility of re-identifying the individual donors, but also because of the specific information about individuals that may be encoded in microbiome-related data. However, we draw attention to circumstances in which bacterial cultures are cultivated from these samples, but exist separately and cannot be linked back to individual donors; our argument of treating human microbiome research samples as ‘human’ samples does not extend to these cases. We also propose that returning individual results in human microbiome research can provide a valuable clinical tool for patient care management, but highlight the need to address how to manage the processes ethically and consider contextual factors that may be unique to human microbiome research.

As we have outlined in the context of probiotics and fecal transplants, the drive for translating human microbiome research into practical applications requires further understanding of how scientific, clinical, political and public interests and concerns intersect and are negotiated. By articulating these ethical challenges, we hope to contribute to the discussion on the ELSI of human microbiome research in ways that can foster more collaborative dialogue between scientists, biobank personnel, research ethics boards, and relevant regulatory agencies for ethical guidance regarding the use of human microbiome research samples and related data.
